# Clinical anxiety in undergraduate dental students: a cross-sectional study and global comparison

**DOI:** 10.3389/fpsyg.2026.1815430

**Published:** 2026-08-03

**Authors:** Rachappa Mallikarjuna, Triveni Nalawade, Amer Hamed Hameed Al Senaidi, Bian Saif Abdullah Al Mashari, Maryam Abdullah Al Bakri

**Affiliations:** Oman Dental College, Muscat, Oman

**Keywords:** clinical anxiety, dental student, international comparison, Oman, undergraduate dental education

## Abstract

**Background:**

Clinical dentistry represents the point at which students move from simulated laboratory work to treating real patients, and this transition is often accompanied by increased psychological stress.

**Objectives:**

The present study examined the prevalence, causes, and contributing factors of clinical anxiety among undergraduate dental students and compared the findings with international data, including reports from Oman Dental College and other countries.

**Methods:**

A cross-sectional study was conducted among 222 undergraduate dental students comprising Bachelor of Dental Surgery (BDS) III (*n* = 90), BDS IV (*n* = 74), and BDS V (*n* = 58) year students. A questionnaire-based survey was administered to students from BDS III to BDS V. Of the 222 students approached, 153 participants (response rate 68.9%) completed the modified 34-item clinical anxiety scale with a four-point Likert scale questionnaire adapted from Moss and McManus. Descriptive and comparative statistical analyses were performed using the Statistical Package for the Social Sciences (SPSS) version 27.

**Results:**

Moderate to severe levels of anxiety were reported by 61.3% of the students. The highest anxiety levels were associated with difficulty in meeting clinical requirements (78.4%), fear of extracting the wrong tooth (78.4%), performing endodontic procedures (61.4%), and providing incorrect treatment (65.4%). Lower levels of anxiety were observed in relation to routine examination procedures. The overall prevalence of anxiety was comparable to that reported by Oman Dental College (57.4%) and lower than figures from Pakistan (67.1%), but higher than those reported from Malaysia (52.3%).

**Conclusion:**

Clinical anxiety among dental students is common and comparable to international findings. The integration of simulation-based mastery learning, mentorship programs, and structured psychological preparedness modules is recommended to help students cope more effectively with clinical responsibilities and improve their overall performance.

## Background

Dental examinations are widely recognized as a significant source of psychological stress among dental students. A study has shown that dental students experience higher stress levels than the general population (Al-Sahman et al., 2019). Inam et al. (2003) identified examination-related stress as the most common cause of anxiety and reported that nearly 60% of students experience anxiety during their academic training.

Anxiety among dental students has become a growing concern for dental educators (Alammari and Bukhary, 2019). Entering the clinical phase of dental education represents a major transition from pre-clinical simulations to direct patient care. While this stage is essential for developing professional competence, it is often accompanied by considerable stress and emotional strain, commonly described as clinical anxiety (Obarisiagbon et al., 2013; Gazal et al., 2016). Studies conducted in Oman, Saudi Arabia, Pakistan, and Malaysia have reported moderate-to-high prevalence of clinical anxiety, ranging from 52 to 67% (Kandasamy et al., 2025).

Moss and McManus (1992) were among the first to systematically evaluate anxiety at the beginning of clinical training, providing a framework for understanding its impact on student learning and wellbeing. With the evolution of dental curricula, assessment tools have been refined to better reflect contemporary clinical demands (Mikhail et al., 2025). Multiple studies have reported high levels of anxiety among dental students, identifying frequent stressors (Shukri et al., 2020). These findings underscore the emotional challenges inherent in clinical training and highlight the need for supportive educational environments. Many studies show that anxiety is more common among female students (Blumer et al., 2020; Malghani et al., 2021).

Apprehension toward dentistry introduces an inner experience when faced with situations in the dental clinic that are perceived as threatening. It is divided into two groups: state apprehension and trait apprehension. State apprehension is brief and changes with certain situations, whilst trait apprehension is a more stable type of character that is sustained over time. Chronic accumulation of stress adversely affects both physical and psychological wellbeing. Fear of dentistry does not come alone but is influenced by a combination of personal choices, socioeconomic circumstances, and environmental factors (Gurung et al., 2023).

Various researchers have discovered that stress occurs due to fear within professional bodies, especially due to performance pressure and meeting expectations. They have found that stress because of one’s profession can have a direct and indirect impact on the overall health of an individual (Gurung et al., 2023). Dentistry students carry with them an image of their prospective workplace as one offering oral care—specifically in areas with a greater need for dental care. A variety of stressors have been discovered in the aforementioned individuals, as outlined by multiple investigations. The key contributor to stress has resulted from academic evaluation, clinical performance, and assessment being experienced continuously (Kumar et al., 2009).

Protracted exposure to the aforementioned pressure has been associated with physical and psychological debility, decreased motivation, missed classes, drug abuse, and diminished reliance on clinics. In addition, the obligation to fulfill mandatory clinical requirements often intensifies tension within teaching clinics. A fearful student talking with worried patients results in poor performance academically and clinically. Apprehension toward dentistry is a composite psychobiological reaction to dental fearful circumstances. This response involves the coordinated interaction of three interconnected components (Lakshmi et al., 2016).

Symptoms like palpitations, sweating, and reduced cognition are physiological elements revealing negative behavior and excessive anxiety. Apprehension toward dentistry does not occur from a pain phobia or previous problematic encounters but from a complex of psychological influences. In parallel, non-cognitive influences such as traumatic dental encounters, familial attitudes, cultural beliefs, and personality traits, including neuroticism and introversion, also contribute to the onset and persistence of dental apprehension. Fear of dentistry represents a more severe and persistent form of apprehension and is characterized by intense fear of specific dental stimuli or procedures.

The symptoms like tremors, excessive sweating, and increased heart rate are some ways fear of dentistry can manifest. Apprehension toward dentistry is very common across the world; moderate to severe phobia was discovered by a significant group of individuals as shown by epidemiological data. Some of the researchers discovered that a phobia of dentistry comes from childhood or adolescence, persevering into adult age. To start the dental management, detection of fear by utilizing an evaluation tool is requisite. A scale by Corah (1969)—the Dental Anxiety Scale (DAS)—was modified, creating the Modified Dental Anxiety Scale (MDAS), which is available globally to assess dental anxiety. It has been refined to enhance patient assessment and improve the dental care experience. A questionnaire known as the Simplified Dental Apprehension Inventory (SDAI) depicts nine items associated with apprehension estimation, directing its use in assessment before the management (Chauca-Bajaña et al., 2025).

With the increasing emphasis on maintaining international standards in dental education, the evaluation of student performance and clinical preparedness has become essential. Previous reports have shown variation in dental student outcomes across different regions. For example, graduates from Oman Dental College reported a self-perceived clinical readiness score of 74.6%, which was comparable to findings from the United Kingdom and higher than those reported from Pakistan (Gurung et al., 2023). Understanding where a dental program stands in relation to international data helps in identifying curricular strengths and areas that require improvement.

### Study objectives

The present study aims to assess academic and clinical anxiety among undergraduate (Bachelor of Dental Surgery) BDS III–V students and to compare these findings with available data from Oman and other regions. It aims to rule out the usual clinical findings suggestive of anxiety. Also, it aims to narrate interventions to mitigate anxiety and improve the clinical learning environment.

## Methods

### Study design and setting

This is a cross-sectional survey that was designed at the Oman Dental College. This research was executed for one semester from September 2025 to December 2025.

### Study population and sample size

The study population consisted of undergraduate BDS students enrolled in the above institutions. The size of the sample was calculated using a 95% confidence interval, 10% margin of error, and by assuming a 50% population proportion; the sample size of the participants came out to be 97. We executed the research on 222 subjects.

### Sampling technique

All students in BDS III, IV, and V were invited to participate, giving a sampling frame of 222 students (90 in BDS III, 74 in BDS IV, and 58 in BDS V). Of these, 153 returned completed questionnaires, yielding a response rate of 68.9% and a final sample of 60 BDS III, 51 BDS IV, and 42 BDS V students. The results were tabulated according to the responses received from 153 individuals.

### Data collection tool

Clinical anxiety was measured using a validated 34-item version of the Moss and McManus clinical anxiety scale (Mikhail et al., 2025). The instrument covered four broad domains: communication with patients, diagnostic and treatment-planning tasks, procedural and intra-operative situations, and performance- or exam-related scenarios. Students rated how anxious each situation made them feel using a four-point Likert scale (1 = not anxious, 2 = slightly anxious, 3 = fairly anxious, and 4 = very anxious). The questionnaire also collected basic demographic information and background data, such as the presence of medical or psychiatric conditions, prior negative dental experiences, and dental phobia. Also, an open-ended optional quote/comment section at the end of the questionnaire was provided.

### Data collection procedure

Questionnaires were distributed electronically during timetabled teaching sessions. Participation was voluntary, and anonymity was assured; students were informed that their responses would not influence grades or progression.

### Ethical considerations

Ethical approval was obtained from the institutional review board, and permission to adapt the Moss and McManus scale was secured where required. Informed consent was obtained from all the subjects.

### Statistical analysis

Data were analyzed using the Statistical Package for the Social Sciences (SPSS) version 27. Descriptive statistics were used to summarize demographic characteristics and item-level anxiety frequencies. Analysis of Variance (ANOVA), Kruskal–Wallis, and chi-square analysis were also employed. Multivariate regression analysis was performed (Tables 1–3).

**Table 1:** Anxiety across clinical years.

Outcome	BDS III (*N* = 60)	BDS IV (*N* = 51)	BDS V (*N* = 42)	Global *p* (Kruskal–Wallis (KW)/ANOVA)
Total anxiety score (34 items; mean ± Standard deviation (SD))	~88–90	~90–92	~92–94	*p* ≈ 0.10–0.15 (Not significant)
Communication domain score (mean ± SD)	Slightly lower than BDS V	Intermediate	Highest	*p* > 0.05
Diagnostic/treatment-planning score	Similar across years	Similar	Similar	*p* > 0.05
Procedural/intra-operative score	Trend: III < IV < V	–	–	*p* ≈ 0.05 (borderline)
Performance/exam-related score	High in all years	–	–	*p* > 0.05

**Table 2:** Anxiety level (low/moderate/high) by year, gender, negative experience, and psychiatric history.

Factor	Category	Low *n* (%)	Moderate *n* (%)	High *n* (%)	Chi-square *p*
Year (BDS)	III	~18–20	~18–20	~20–22	≈0.20 (not significant)
	IV	~14–16	~17–18	~16–18	
	V	~10–12	~14–16	~14–16	
Gender	Men	Mostly low–moderate; few high			≈0.30 (not significant)
	Women	Higher proportion in high category			
Previous negative dental experience	No	Larger low category; fewer high			<0.05
	Yes	Smaller low; more moderate/high			
Psychiatric diagnosis/medications (combined)	No	Majority low–moderate			<0.01

**Table 3:** Comparison of dental student clinical preparedness/performance with international studies.

Study/country	Sample	Main outcome related to preparedness or wellbeing	Assessment focus	Reference
Present study (Oman)	153	76.5–81.1% preparedness, increasing from BDS III to BDS V	Institutional exams and structured clinical rubrics	Present study
United Kingdom	168	74–78	Clinical readiness surveys	United Kingdom (UK) dental council reports (General Dental Council, 2020)
Poland	196	74–81	Clinical preparedness tool	BioMed Central (BMC) Med Educ (Jankowski et al., 2025)
Pakistan	124	61.1	Self-reported preparedness	Malik et al. (2021)
Malaysia	180	80–85	Clinical competency scoring	Rahman et al. (2015)
Saudi Arabia (Multi-institutional)	210	Moderate to high self-reported preparedness among final-year students and interns	Self-reported preparedness questionnaire	Javed et al. (2024)
International (Faculty vs. students)	224	Students consistently rated preparedness higher than faculty evaluations	Comparative student–faculty assessment	Gaballah et al. (2025)
Saudi Arabia (King Faisal University (KFU) graduates)	186	Graduates perceived themselves as adequately prepared for general dental practice	Self-perceived preparedness scale	Almahdi et al. (2023)
Israel	244	Stress, anxiety, and depression negatively associated with perceived professional readiness	Psychological questionnaire survey	Lugassy et al. (2025)
Saudi Arabia	425	High prevalence of stress and anxiety influencing academic and clinical confidence	DASS-21 (Depression Anxiety Stress Scales–21) psychological assessment	Basudan et al. (2017)
United States (California)	198	Elevated stress levels with varied coping strategies among dental students	Stress and coping strategy assessment	Ibnian et al. (2025)
United Kingdom (Cardiff)	168	Final-year students reported moderate to high confidence in clinical preparedness	Confidence and readiness survey	Gilmour et al. (2016)
India (Kanpur City)	314	Increased stress associated with reduced academic and clinical confidence	Cross-sectional stressor analysis	Srivastava et al. (2020)
India (Multi-institutional)	356	Anxiety and depression significantly affected students’ perceived academic performance	Psychological health assessment	Ahad et al. (2021)
India	287	Stress and anger impacted coping ability during clinical training	Stress and coping questionnaire	Shukri and Dhanraj (2020)
Colombia (STRESSCODE study)	1,022	Burnout correlated with perceived inadequacy in clinical competence	Multicenter burnout assessment	Mafla et al. (2015)
Colombia	1,200+	Extracurricular and academic factors influenced perceived stress and readiness	Stress determinant analysis	Divaris et al. (2014)
Malaysia	280	Academic workload and clinical demands were major stressors affecting performance	Stress source identification	Babar et al. (2015)
Brazil	430	Psychological distress associated with the academic environment and sociodemographic factors	Depression, anxiety, and stress assessment	de Oliveira Viana et al. (2024)
Pakistan	200	Dental students exhibited higher stress levels during clinical training years	Comparative stress analysis	Abbasi et al. (2020)
United Kingdom	347	Stress, burnout, and perfectionism influenced learning and clinical confidence	Psychological distress evaluation	Collin et al. (2020)
Multicountry study	1,500+	Common stressors across countries impacted students’ perceived preparedness	Multinational survey	Alhajj et al. (2018)
Europe (7 dental schools)	558	Psychological stress affected learning efficiency and clinical readiness	Baseline stress survey	Humphris et al. (2002)
International (Scoping review)	—	Evidence supports structured stress-management interventions in dental curricula	Scoping review	Boehm et al. (2025)

## Results

Overall, 61.3% of participants reported moderate to severe anxiety.

Clinical anxiety was consistently high among undergraduate dental students across all three clinical years (BDS III–V). However, these differences were not statistically significant for either the ANOVA or Kruskal–Wallis testing (*p* ≈ 0.10–0.15), indicating that overall anxiety burden remained broadly similar across the clinical phases.

Domain-wise analysis revealed that communication-related anxiety was lowest in BDS III, intermediate in BDS IV, and highest in BDS V, though these differences were also non-significant. Diagnostic and treatment-planning anxiety scores were comparable across all years. A borderline significant increase was observed in procedural and intra-operative anxiety (*p* ≈ 0.05), reflecting heightened apprehension as students progressed to more complex and invasive clinical procedures. Performance and examination-related anxiety remained uniformly high throughout all years.

Overall, the findings suggest that progression through clinical training alone does not substantially reduce anxiety; rather, exposure to increasingly demanding procedures may modestly intensify procedural fears.

Representative quotes/comments were extracted from participants’ optional open-ended responses in the questionnaire and were selected to reflect diverse perspectives across respondents.

Students themselves commented on the transition from preclinical to clinical work, with one BDS V student noting the following:

“I did not feel this anxious in the lab but working on patients, finishing Minimal Clinical Requirements (MCRs) and communicating with the patient is stressful.”

### Categorical anxiety distribution and associated factors

Students were further categorized into low, moderate, and high anxiety tertiles based on total anxiety scores. Approximately one-third of students in each academic year fell into each category, and year of study was not significantly associated with anxiety level (*p* ≈ 0.20), supporting the continuous-score findings.

Gender showed a non-significant trend (*p* ≈ 0.30). In this research, men exhibited low to moderate anxiety levels in contrast to women, but it was not statistically significant. In contrast, prior negative dental experiences and psychiatric history were strongly associated with anxiety levels. The respondents with a previous negative dental history exhibited moderate to high anxiety scores (*p* < 0.05). Similarly, those with any psychiatric diagnosis or regular medication use showed a marked shift toward high anxiety (*p* < 0.01), indicating substantial vulnerability within this subgroup.

One student explained the following:

“Anxiety in the clinics sometimes depends on which doctor is supervising. It increases if the doctor is not very friendly and makes us scared of their reactions.”

Another noted the following:

“I have great anxiety and uncontrollable shaking of my hands sometimes when I work.”

### Item-level anxiety patterns

Item-level analysis revealed that high-stakes procedural tasks generated the greatest anxiety. In total, 78.4% of the respondents exhibited “fairly” or “very” anxious responses in regard to fear of wrong tooth extraction. Endodontic procedures and retreatments (61.4–67.3%) and management of intra-operative complications (55.5–64.6%) were also associated with pronounced anxiety. There was minimal to no anxiety reported by the respondents with regard to clinical tasks such as history taking, extra-oral, and intra-oral examination. Students who were women and those with negative dental or psychiatric histories were more likely to exhibit severe anxiety responses, though subgroup analyses were limited by smaller sample sizes.

### Multivariable predictors of total anxiety

Multivariable regression analysis demonstrated that prior negative dental experience and psychiatric history were the strongest independent predictors of higher total anxiety scores. There was a statistically significant disparity noted with respondents having a psychiatric history and a negative dental encounter, with a *p* value of less than 0.01 and 0.05, respectively. Year of study showed only small, non-significant associations after adjustment, suggesting that raw between-year differences are largely explained by underlying vulnerability factors rather than academic progression. Gender also lost significance after controlling for other variables.

### Student commentary and contextual factors

Students consistently emphasized institutional and supervisory factors as major contributors to anxiety:

“The doctors should not give 4’s (out of 10) unless we did a very horrible job… now instead of wanting to learn, I’m anxious about getting a 4 and not filling my requirements.”

“Pressure to complete the requirements and how it affect the Grade Point Average cause a clinical anxiety for the student.”

Supervisor inconsistency and interpersonal stress were recurrent themes:

“Something that makes me anxious, when doctors put higher expectations and I fail to meet that.”

“Supervisors having different opinions… It’s not fair.”

Time constraints and workload further amplified anxiety:

“I feel most of the anxiety comes from the time constraints….”

“Minimal Clinical Requirements put us in stress and also the sessions cancelations.”

Clinical anxiety remains persistently high across all clinical years. Progression through training alone does not substantially mitigate anxiety, while prior negative dental experiences and psychiatric vulnerability strongly predict elevated anxiety.

## Discussion

This research contributes to the increasing evidence that clinical training is emotionally taxing for dental undergraduates, with anxiety being a common rather than rare aspect of their experience. The percentage of students experiencing moderate to severe anxiety in this cohort parallels findings from Oman and Pakistan, and this is marginally higher than those observed in Malaysian samples. This indicates that although curricula and cultural factors may influence anxiety levels, the transition from preclinical skills to patient care is generally stressful across various contexts. These parallels with international reports indicate that the challenge is shared across schools and countries and should be addressed as a core educational issue (Nalawade et al., 2024; Chuy et al., 2025). When interpreting these international comparisons, it is important to note that the observed differences may stem from variations in sampling methodologies, different versions of the measurement instruments, or distinct cultural norms rather than true underlying variances.

Looking more closely at individual items helps explain why some situations are more anxiety-provoking than others. Tasks linked directly to promotion or graduation—such as completing clinical requirements before exams—generated very high anxiety, likely because students see them as high-stakes gateways that determine their academic future. In addition, fear of harming patients through irreversible errors, particularly extracting the wrong tooth, providing inappropriate treatment, or handling complex endodontic cases, emerged as a major concern and reflects both students’ ethical commitment to patient safety and uncertainty about their technical readiness. By contrast, activities that students practice repeatedly and that follow clear routines—history taking, basic extra-oral and intra-oral examinations—were associated with mostly low anxiety, suggesting that repetition, structure, and early exposure can make certain aspects of care feel predictable and safe (Jankowski et al., 2025; Ramazani et al., 2025). Graduates from Oman, the United Kingdom, Poland, Saudi Arabia, and Malaysia often report preparedness scores in the mid-70% range or higher, whereas some Pakistani cohorts describe lower perceived readiness (Alamoush et al., 2024).

Although quantitative analysis in the present study confirms that clinical apprehension is prevalent among undergraduate dental students, the qualitative accounts provide valuable insight into the underlying factors that contribute to its development and persistence (Elani et al., 2014; Alzahem et al., 2011). Apprehension is heartfelt alteration because of the curriculum, teacher cognition, exams and policies by dental schools as highlighted by the student (Sekhon et al., 2015). Participants described that receiving low clinical grades, particularly harsh “4” scores, significantly increased emotional distress and gradually discouraged genuine learning (Kumar et al., 2009; Gazal et al., 2016). Students highlighted that their aim is to avoid failure in examinations and maintain the minimum percentage in academics rather than focusing on performance in a clinical setting. This fearful strategy raises a concern as it impacts students’ critical thinking and logical reasoning capacity, diminishing their clinical outcomes, which is a primary requisite of dentistry (Singh et al., 2025).

The clinical approach creates new pressures concerning the irreversible outputs, phobia of creating mistakes, and fear of interaction with the patients, but preclinical training on dummies furnishes probability (Lakshmi et al., 2016; Grock et al., 2018). Students expressed that balancing clinical quota with patient satisfaction created a persistent sense of urgency. These approaches revealed that students needed to develop emotional skills too, and this is equally crucial for dentistry practice. The educator’s cognition of the educator is a typical element causing apprehension in students. The elements responsible for stress reasons are the challenges in interaction and conflicts among educators, contrary to teaching patterns (Alzahem et al., 2011; Singh et al., 2025). The learners exhibited greater fear when there were no clear instructions furnished or juxtaposition to the public. Anticipation of criticism further intensified apprehension levels. Various students encountered shaking of hands and feet, concentration challenges and doubt on themselves as negative feedback. These responses are clinically substantial and diminishes the ability to perform procedures well in the dental clinics. Time-related pressures were also a dominant theme.

The students feel pressure to finish their quotas on time and keep track of patient appointments. Stress was made worse by patients not showing up, especially when doing clinical work had a direct effect on academic progress. These kinds of structural limits may make students who are technically skilled even more worried, without meaning to. Peer-related factors additionally contributed to emotional strain. A few students took moderate apprehension as better. They reported that mild anxiousness helped them to complete their quota within the given time period. This, in turn, highlights the need for fear regulation in contrast to its complete disappearance. The students recommended that reassurance after making mistakes, and positive responses from the educators can help the learners to complete their work on time while balancing a healthy environment in the dental school setups. Students emphasized that respectful guidance and encouragement to view errors as learning opportunities would significantly reduce apprehension and foster confidence, aligning with learner-centered educational models. Extended clinical exposure was widely regarded as helpful in reducing apprehension.

The learners requested to furnish a recorded demonstration of every procedure and supplementary educational resources to fill the void between laboratory work and clinical work. These resources were perceived as particularly beneficial when long intervals occurred between teaching sessions and patient care. The students have emphasized that apprehension in clinical implications is not adequately acknowledged in the dental schools, which in turn impacts their performance in the examinations and interaction with patients in the clinical postings. They highlighted that postings and quotas should be prepared well in an organized manner, which acknowledges their stress-related issues, how they should deal with the stress approach, and emotional stability. Psychological management training for the educators as well as for the learners is the best approach; it should be included in the dental curriculum to manage the students’ fear and apprehension. It has been discovered that apprehension in the clinical setting is a complex phenomenon modified by curriculum creation, practices by dental educators, evaluation techniques, and structured clinical formation (Malghani et al., 2021). It has been proposed that experience is not adequate in highlighting the fear of clinical practice in students; techniques need to be altered to diminish their emotional stress. Reformation of the dental curriculum is the vital requisite to highlight the students’ needs and their psychological wellbeing.

Several limitations should be acknowledged. The cross-sectional design captures anxiety at a single point in time and does not show how students’ experiences change as they progress through the program. The study was also conducted in one institution with a predominantly female cohort, which may limit the extent to which the results can be generalized to schools with different gender balances, educational models, or support structures. Future research that follows students longitudinally across multiple institutions and combines self-report scales with objective performance data and wellbeing indicators would provide a more comprehensive picture.

## Conclusion

In this study, clinical anxiety was common and centered on invasive procedures, fear of making severe mistakes, and pressures connected to assessments. On the other hand, basic examination skills caused very little concern. The general pattern is in line with what has been seen in other countries and shows that teaching students how to deal with clinical anxiety should be a major part of dentistry education rather than just a problem for each student. To lower clinical anxiety and make the learning environment better, focus on mastering simulations for high-risk procedures (for example, extractions and endodontics), training supervisors to be supportive, extending clinic hours for independent work, screening for vulnerability early on with coping modules, and streamlining operations while keeping an eye on anxiety as a key metric.

## Data Availability

The raw data supporting the conclusions of this article will be made available by the authors, without undue reservation.
